# Effects of crab shell extract as a coagulant on the textural and sensorial properties of tofu (soybean curd)

**DOI:** 10.1002/fsn3.837

**Published:** 2019-01-27

**Authors:** Joon‐Young Jun, Min‐Jeong Jung, In‐Hak Jeong, Gwang‐Woo Kim, Jae‐Man Sim, Si‐Youl Nam, Byoung‐Mok Kim

**Affiliations:** ^1^ Division of Strategic Food Research Korea Food Research Institute Gangneung Korea; ^2^ Department of Marine Food Science and Technology Gangneung‐Wonju National University Gangneung Korea; ^3^ Center of Marine Biotechnology Gangneung Science and Industry Promotion Agency Gangneung Korea; ^4^ Donglim Food Co. Ltd. Gangneung Korea

**Keywords:** calcium, *Chionoecetes japonicus*, coagulant, crab shell, sensorial acceptability, texture, tofu

## Abstract

To find an economic use of red snow crab (*Chionoecetes japonicus*) shell waste, we focused on its high mineral content. To evaluate its usability as a coagulant for tofu making, the effects of the crab shell extracts on the textural and sensorial properties of the tofu samples were investigated. The crab shell powder (CSP) and ash (CSA) were used for their extract preparation, and 1%–5% acetic acid treatment led to an abundance of calcium in the resulting extracts. The tofu yields of all the acetic acid extracts were comparable with those of the commercial coagulants MgCl_2_ and glucono‐δ‐lactone (GDL). Furthermore, the results for the textural attributes and sensorial acceptability demonstrated that either the extract from CSP prepared with 3% acetic acid or the extracts from CSA prepared with 1% or 3% acetic acid could be used as coagulants, because all the values of the extracts were statistically equivalent to those of the MgCl_2_ and GDL (*p *<* *0.05).

## 1 INTRODUCTION

In East Asia, tofu is a very popular food and is considered to be an excellent protein source due to its high‐quality protein and digestibility (Cheng, Shimizu, & Kimura, 2005; No & Meyers, 2004). Tofu is made from soybean milk with heat treatment and coagulants; glycine and β‐conglycinin form a protein gel through their interactions (hydrogen, hydrophobic, and disulfide bonds) during heat denaturation, and divalent ion coagulants play a key role in making ionic bridges between the proteins for constructing the three‐dimensional network structure of tofu (Utsumi & Kinsella, 1985; Yoon & Kim, 2007). There are various factors affecting tofu quality, such as soybean cultivar (protein ratio), processing conditions (brix of soymilk, heating, and mixing) and coagulant; among these, the type of coagulant and its concentration are the most crucial factors for the textural property of tofu (Johnson & Wilson, 1984). As coagulants, calcium or magnesium salts and glucono‐δ‐lactone (GDL) have been primarily used. Since consumers consider the relationship between food and health to be important, demand for chemical‐free natural products has increased, and the development of alternative natural additives is required (Hwang, Yoon, Kang, Sim, & Shin, 2013; Kim et al., 2008).

According to the global production statistics of the Food and Agriculture Organization of the United Nations, annual crab production has risen consistently for the past 10 years, increasing from 159 million tons in 2007 to 202 million tons in 2016 (FAO, 2018). With this increase, the growing production of shellfish by‐products has become a concern. In fact, the worldwide production of shellfish waste has been highlighted, as it may pose an environmental hazard (Ibrahim, Salama, & El‐Banna, 1999). Although there are species differences, a vast quantity of crab shell waste is generated from crab processing; the red snow crab (*Chionoecetes japonicus*) is the most utilized industrial species in the Republic of Korea, and the crab shell accounts for an approximately 65% of the whole fish. Since the biodegradation of such crab shells is very slow and the shell industrial use is low, this unnecessary waste generation has become a concern in the crab industry (Shahidi & Synowieck, 1991). Crab shells contain various nutritional and valuable components, including proteins, minerals (rich in calcium), chitins, and carotenoids, and they have been recycled and valorized as nutraceutical chitin/chitosan, animal foods, natural pigments, etc. (Murphy et al., 2003). Nevertheless, these uses do not provide sufficient alternatives for all crab shell waste. A novel approach for a better economic use might reduce the concern for industrial waste and offer new business opportunities.

In this regard, we focused on the mineral content of the red snow crab shell to utilize as a coagulant for tofu. The conditions for preparation of a calcium‐rich extract from the crab shell and their effects on the yield, and textural and sensorial properties of the tofu were investigated.

## 2 MATERIALS AND METHODS

### 2.1 Materials

The carapaces of red snow crabs (*Chionoecetes japonicus*), which were obtained from the Sung‐Jin Trading Co. Ltd. (Sokcho, Republic of Korea), were used for the preparation of crab shell extracts. Two commercial coagulants (food grade), CaSO_4_ and glucono‐δ‐lactone (GDL), were purchased from the ES Food Ingredient Co. Ltd. (Gunpo, Republic of Korea). A commercial coagulant MgCl_2_ (food grade) was purchased from the Ronic Co. (Paju, Republic of Korea). The soybeans (*Glycine max*) used for the preparation of tofu were purchased from a local market.

### 2.2 Preparation of crab shell extract

The carapaces were dried in the sun (until ca. 15% moisture content), crashed, sieved below 500 μm (crab shell powder, CSP), and ashed at 650°C for 12 hr (crab shell ash, CSA). Each 20 g of the CSP and CSA were extracted with 800 ml of 0%, 1%, 3%, or 5% acetic acid at room temperature for 12 hr and filtered using a filter paper (≤5 μm). The filtrates were freeze‐dried and weighed to calculate the yields of the extracts. In total, eight crab shell extracts were prepared: the extract from CSP with 0% acetic acid (deionized water, DW), CSP‐A0; the three extracts from CSP with 1%, 3%, or 5% acetic acid, CSP‐A1, CSP‐A3, and CSP‐A5; the extract from CSA with 0% acetic acid (DW), CSA‐A0; the three extracts from CSA with 1%, 3%, or 5% acetic acid, CSA‐A1, CSA‐A3, and CSA‐A5. The yields of the extracts were expressed as g per 100 g of the CSP or CSA.

### 2.3 Determination of proximate composition, acetic acid, and pH

To specify the chemical properties of the CSP, CSA, and their extracts, the proximate composition, acetic acid content, and pH value were determined. The moisture, ash, crude protein, and crude lipid contents were measured according to the AOAC method (AOAC, 2005). The acetic acid contents were analyzed using a HPLC (Agilent 1,200; Agilent Technologies, Santa Clara, CA, USA) equipped with a diode array detector (Jun, Lim, Lee, Kim, & Jeong, 2016). Each of the samples was dissolved in 8 mm Na_2_SO_4_ adjusted pH to 2.8 using H_2_SO_4_ (eluent) and filtered using a 0.2 μm polytetrafluoroethylene syringe filter unit. A 10 μl aliquot of the sample was injected, and the analysis was performed with a μBondapack^™^ C18 column (10 μm, 3.9 × 300 mm; Waters Co., Milford, MA, USA) under the following conditions: 35°C of column temperature, 1 ml/min of flow rate, and 210 nm of wavelength. The acetic acid in the sample was identified and calculated by comparison with the retention time and area of acetic acid as a standard. For pH determination, each 1 g sample was mixed with 20 ml DW, and the pH was determined using a pH meter (SevenEasy S20K; Metteler Toledo International Inc., Columbus, OH, USA).

### 2.4 Determination of minerals

To determine the mineral contents (Na, K, Ca, Mg, Mn, Cu, Fe, and Zn) in the CSP, CSA and their extracts, each 0.5 g sample was digested with 15 ml HNO_3_ using a microwave digestion system (Mars 5; CEM Co., Matthews, NC, USA). After an appropriate dilution with DW, the mineral contents in the digested sample were analyzed by an air‐acetylene flame technique using an atomic absorption spectrophotometer (PK‐300; Perkin Elmer Inc., Waltham, MA, USA).

### 2.5 Preparation of tofu

Tofu was prepared by the method described by No and Meyers (2004) with slight modification. A 125 g of the soybean was soaked in 10°C tap water for 36 hr and drained. The soaked soybeans were mixed with 750 ml tap water and blended. The mash was filtered with a cotton cloth and pressed. The brix of soymilk was adjusted to ca. 10° with tap water in a stainless steel tank and heated at 90°C for 15 min with constant stirring. Then, each of the eight crab shell extracts or three commercial coagulants dissolved in DW was added to a final concentration of 0.16% (w/v) and stirred for 5 min gently. As a solvent control, 0.08% (v/v) acetic acid alone was added. After cooling at room temperature for 1 hr, the curd was separated into tofu and waste liquid by filtering through a two‐layered gauze.

The total weight of the tofu and the total volume of the waste liquid were determined to calculate the yield and protein coagulation rate; in the case of the waste liquid, the protein content was measured according to the AOAC method (AOAC, 2005). The yield was expressed as g per 100 g of raw soybean, and the protein coagulation rate was calculated using the following equation: protein coagulation rate (%) = (1 – [total protein in the waste liquid/total protein in the soymilk]) × 100.

### 2.6 Determination of texture

The textural properties of the tofu samples were characterized using a texture analyzer (TAXT plus; Stable Micro System, Goldalming, UK) fitted with a 5 kg load cell. Each of the tofu samples was cut into a cube (1.5 × 1.5 × 1.5 cm) and compressed twice until 50% deformation with a 36 mm diameter cylindrical probe (P/36R). The test speed was set at 1 mm/s, and the hardness, springiness, cohesiveness, and gumminess were measured (Bourne, 2002).

### 2.7 Sensory test

The acceptability tests of the tofu samples were conducted according to the method of Kim et al. (2016). Before testing, all samples were stored at 4°C for 1 hr and served to 10 untrained panelists (five men and five women: 20–40) who were familiar with eating tofu. All samples were coded and placed in a randomized arrangement. The acceptance questions (appearance, color, flavor, taste, and overall acceptance) were asked using a nine‐point hedonic scale: one represented “extreme dislike” and nine represented “like very much.”

### 2.8 Data analysis

All the data, except for the mineral content, were expressed as the mean ± standard deviation (*SD*) and were statistically assessed by a one‐way ANOVA test; a significant difference (*p *<* *0.05) in the means was identified by Tukey's test using the SPSS program (IBM, Armonk, NY, USA). The data of the mineral content were expressed as the mean in duplicate determinations, because all the *SD* values were <5% of the mean values.

## 3 RESULTS AND DISCUSSION

### 3.1 The yields of the crab shell extracts

In general, crustacean shells contain 30%–40% protein, 30%–50% calcium carbonate, and 20%–30% chitin, though there are variations in species and seasons (Arbia, Arbia, Adour, & Amrane, 2013); the mineral content tended to be higher in hard shell crustaceans than in soft shell crustaceans (Ibrahim et al., 1999; Lage‐Yusty, Vilasoa‐Martínez, Álvarez‐Pérez, & López‐Hernández, 2011). The high calcium content in the crab shell led to use as a coagulant. Ashing the crab shell might facilitate mineral recovery, but the advantage of eliminating organic components from the crab shell is unpredictable on tofu quality; in some cases, the addition of chitosan increased the shelf life of tofu due to its antimicrobial activity (Chang, Lin, & Chen 2003; No & Meyers, 2004). For this reason, the crab shell extracts were prepared from both the two raw materials CSP and its ash (CSA).

The yields of the crab shell extracts with 0%–5% acetic acid are shown in Table 1. The 0% acetic acid (DW) yielded 8.1 and 18.9 g extracts from 100 g of the CSP and CSA, respectively. By contrast, the yield was significantly increased with treatment of acetic acid in both cases of the raw materials (*p* < 0.05). From CSP, 1% to 5% acetic acid treatments yielded ranging from 57.9% to 64.9%; in particular, the two extracts from CSA with either 3% or 5% acetic acid showed the highest yields (159.2% and 170.2%, respectively) among all the extracts (*p *<* *0.05). The calcium recovery of organic acids such as acetic acid and lactic acid have been demonstrated to be excellent (Jang, Park, & Jeong, 2005).

**Table 1:** The yields of the acetic acid extracts from the crab (*Chionoecetes japonicus*) shell powder and ash

	Acetic acid conc. (%)	Yield (g/100 g, dry)
CSP^a^	0	8.1 ± 1.2^c,^ c
	1	57.9 ± 2.3^b^
	3	68.1 ± 2.7^b^
	5	64.9 ± 3.8^b^
CSA^b^	0	18.9 ± 2.6^c^
	1	66.2 ± 4.4^b^
	3	159.2 ± 8.9^a^
	5	170.2 ± 8.7^a^

*Notes*

Data expressed as the mean ± *SD* in triplicate determinations.

a Crab shell powder.

b Crab shell ash.

c The different small letters indicate significantly different values in the vertical column (*p *<* *0.05).

### 3.2 The chemical properties of the crab shell extracts

Table 2 shows the proximate compositions, acetic acid contents, and pH values of the CSP, CSA, and their extracts. The CSP was composed of 35.7% ash, 25.0% crude protein and 0.2% crude lipid, while the CSA consisted of mostly ash (98.2%). The proximate compositions of the two extracts from CSP or CSA with 0% acetic acid (DW) were similar with each of the raw materials. In contrast, all the other extracts prepared with acetic acid consisted of 33.9%–44.2% ash and 41.2%–61.9% acetic acid. Interestingly, the protein contents in the extracts from CSP with acetic acid decreased to the values below 2.0%, indicating that the protein in the CSP could be eliminated effectively by the acetic acid treatment. However, a preliminary qualitative analysis using thin‐layer chromatography indicated no existence of chitosan in all the acetic acid extracts from CSP (data not shown).

**Table 2:** Chemical properties of the raw materials and their extracts prepared with 0%–5% acetic acid

	Moisture (%)	Ash (%)	Crude protein (%)	Crude lipid (%)	Acetic acid (%)	pH
CSP^a^	11.9 ± 0.5	35.7 ± 3.2	25.0 ± 1.2	0.2 ± 0.1	Nt^e^	8.9 ± 0.1
CSA^b^	2.1 ± 0.7	98.2 ± 2.3	Nd^f^	Nd	Nt	12.3 ± 0.1
CSP‐A0 c	13.5 ± 2.1^ab,^ g	39.0 ± 1.4^bcd^	32.7 ± 1.2^a^	0.3 ± 0.1^b^	4.8 ± 0.2^d^	8.5 ± 0.0^b^
CSP‐A1^c^	11.1 ± 1.1^bc^	39.8 ± 4.1^bcd^	1.9 ± 0.3^b^	0.7 ± 0.2^a^	47.4 ± 3.4^bc^	4.4 ± 0.0^d^
CSP‐A3^c^	14.6 ± 2.1^ab^	44.2 ± 1.7^b^	1.6 ± 0.2^b^	0.6 ± 0.1^a^	41.2 ± 4.5^c^	4.3 ± 0.0^d^
CSP‐A5^c^	15.7 ± 0.1^a^	43.7 ± 2.7^b^	1.3 ± 0.1^b^	0.3 ± 0.1^b^	44.0 ± 2.8^bc^	4.1 ± 0.0^d^
CSA‐A0^d^	7.3 ± 1.1^cd^	87.6 ± 3.6^a^	Nd	Nd	Nd	11.4 ± 0.1^a^
CSA‐A1^d^	5.8 ± 1.2^d^	41.2 ± 0.2^bc^	Nd	Nd	48.8 ± 1.1^b^	10.9 ± 0.0^a^
CSA‐A3^d^	6.0 ± 1.0^d^	35.3 ± 1.3 ^cd^	Nd	Nd	56.3 ± 1.8^a^	6.0 ± 0.1^c^
CSA‐A5^d^	10.8 ± 2.1^bc^	33.9 ± 2.7^d^	Nd	Nd	61.9 ± 1.0^a^	4.7 ± 0.0^d^

*Notes*

Data expressed as the mean ± *SD* in triplicate determinations.

a Crab shell powder.

b Crab shell ash.

c The extracts from CSP with 0%–5% acetic acid.

d The extracts from CSA with 0%–5% acetic acid.

e Not tested.

f Not detected.

g The different small letters indicate significantly different values in each the vertical column (*p *<* *0.05).

In the case of the acetic acid extracts from CSA, the ash content tended to decrease with increasing acetic acid concentrations and relatively increased acetic acid contents in the extracts. The pH values of CSP and CSA were determined to be 8.9 and 12.3, respectively. All the acetic acid extracts from CSP showed an acidic pH (near pH 4), whereas the pH of CSA‐A1 was a basic. The increase in the pH values was also observed in a report conducted by Shim and Kim (1997) who investigated the calcium ionization effect of organic acids from eggshells. During ashing the crab shell, a thermal decomposition of CaCO_3_ can occur, it is changed to a CaO form and is converted to Ca (OH)_2_ when dissolved in water (Kim, Kim, Kim, & Kim, 1997).

### 3.3 Mineral compositions in the crab shell extracts

To evaluate the mineral recovery of acetic acid from the two raw materials, the mineral compositions of the eight crab shell extracts were analyzed (Table 3). Between the CSP and CSA, the total mineral content was higher in the latter, but the composition ratios of the total minerals were not different. In both the raw materials, calcium was the most abundant (above ca. 89%), followed by magnesium and sodium. According to the extraction solvents, the use of DW increased sodium and potassium in the extracts, whereas acetic acid recovered the most minerals from the raw materials intact, which were rich in calcium, but it was regardless of the acetic acid concentrations (1%–5%) tested in this study.

**Table 3:** Mineral compositions of the raw materials and their extracts prepared with 0%–5% acetic acid

	Macro mineral (mg/g, dry)					Micro mineral (μg/g, dry)			Total (mg/g, dry)
	Na	K	Ca	Mg	Mn	Cu	Fe	Zn	
CSP^a^	5.8 (3.06^e^)	2.1 (1.11)	169.0 (89.28)	10.8 (5.71)	1.4 (0.74)	43 (0.02)	175 (0.09)	19 (0.01)	189.3
CSA^b^	11.1 (2.07)	2.0 (0.37)	493.1 (91.81)	26.9 (5.01)	3.6 (0.67)	87 (0.02)	230 (0.04)	39 (0.01)	537.1
CSP‐A0^c^	112.7 (51.39)	34.8 (15.87)	62.1 (28.32)	20.1 (0.96)	4.1 (1.87)	154 (0.07)	218 (0.10)	12 (0.01)	219.3
CSP‐A1^c^	8.5 (3.55)	5.1 (2.13)	205.2 (85.75)	14.5 (6.06)	5.7 (2.38)	66 (0.03)	196 (0.08)	3 (0.00)	239.3
CSP‐A3^c^	6.8 (2.72)	2.8 (1.12)	220.0 (88.14)	12.8 (5.13)	6.8 (2.72)	78 (0.03)	319 (0.13)	16 (0.01)	249.6
CSP‐A5^c^	9.4 (3.85)	2.8 (1.15)	211.4 (86.57)	12.3 (5.04)	7.8 (3.19)	85 (0.03)	313 (0.13)	13 (0.01)	244.2
CSA‐A0^d^	75.8 (18.51)	18.1 (4.42)	310.2 (75.73)	0.4 (0.10)	9.9 (2.42)	117 (0.03)	361 (0.09)	3 (0.00)	409.6
CSA‐A1^d^	12.4 (4.89)	3.6 (1.42)	226.1 (89.09)	0.4 (0.16)	10.8 (4.26)	131 (0.05)	404 (0.16)	2 (0.00)	253.8
CSA‐A3^d^	5.8 (2.53)	2.4 (1.05)	193.1 (84.21)	16.4 (7.15)	11.1 (4.84)	148 (0.06)	419 (0.18)	6 (0.00)	229.3
CSA‐A5^d^	5.3 (2.22)	2.3 (0.96)	202.7 (84.81)	16.0 (6.69)	12.0 (5.02)	166 (0.07)	471 (0.20)	14 (0.01)	239.0

*Notes*

Data expressed as the mean in duplicate and all the standard deviation were <5% of the mean values.

a Crab shell powder.

b Crab shell ash.

c The extracts from CSP with 0%–5% acetic acid.

d The extracts from CSA with 0%–5% acetic acid.

e Percent ratio in the total minerals detected.

### 3.4 The moisture contents and yields of the tofu samples

The moisture contents, yields, and protein coagulation rates of the tofu samples with the eight crab shell extracts and commercial coagulants are listed in Table 4. The soymilk was not coagulated with the two DW extracts (CSP‐A0 and CSA‐A0) (Supporting information Figure S1), indicating that the raw CSP or CSA alone could not be utilized as a coagulant for tofu making; this phenomenon might be associated with the divalent ion concentrations, pH or the both, additionally, the effect of anion form on the protein gel network have been reported (Obatolu, 2008). By contrast, the tofu was successfully prepared with 0.08% acetic acid alone (a solvent control); the added concentration was based on the average of the acetic acid contents in all the acetic acid extracts from CSP and CSA. Lu, Carter, and Chung (1980) revealed that pH is more important than divalent ions during soymilk coagulation, which is recommended to be near pH 6. This value was in agreement with the average pH (6.2) of the waste liquids determined in this study during gel formation (data not shown).

**Table 4:** The moisture contents, yields, and protein coagulation rates of the tofu samples coagulated with the crab shell extracts and commercial coagulants

Coagulant	Moisture content (%)	Tofu yield (g/100 g, wet)	Protein coagulation (%)
MgCl_2_ a	76.5 ± 1.2^b,^ d	241.4 ± 4.9^b^	90.9 ± 3.9^a^
CaSO_4_ a	78.4 ± 0.7^ab^	146.0 ± 14.0^c^	70.4 ± 6.3^b^
GDL^a^	77.4 ± 1.6^b^	240.4 ± 5.6^b^	88.3 ± 1.0^a^
Acetic acid	81.8 ± 1.8^a^	293.6 ± 24.5^a^	85.3 ± 2.3^a^
CSP‐A1^b^	76.8 ± 2.1^b^	228.9 ± 6.9^b^	88.5 ± 3.3^a^
CSP‐A3^b^	77.2 ± 0.4^b^	240.6 ± 10.4^b^	89.2 ± 0.7^a^
CSP‐A5^b^	76.9 ± 0.7^b^	235.8 ± 19.5^b^	90.3 ± 3.0^a^
CSA‐A1^c^	77.3 ± 1.1^b^	240.8 ± 10.7^b^	88.8 ± 6.4^a^
CSA‐A3^c^	77.1 ± 0.7^b^	244.8 ± 5.5^b^	90.4 ± 3.0^a^
CSA‐A5^c^	76.8 ± 0.4^b^	240.5 ± 11.9^b^	88.2 ± 2.2^a^

*Notes*

Data expressed as the mean ± *SD* in triplicate determinations.

a Commercial coagulants.

b The extracts from CSP with 1%–5% acetic acid.

c The extracts from CSA with 1%–5% acetic acid.

d The different small letters indicate significantly different values in each the vertical column (*p *<* *0.05).

The moisture content of the tofu with 0.08% acetic acid was significantly higher than those of the tofu samples with all the acetic acid extracts including MgCl_2_ and GDL (*p *<* *0.05). The yield of tofu was the highest with 0.08% acetic acid, while it was the lowest with CaSO_4_ (*p *<* *0.05). In both the yields and protein coagulation rates, all the acetic acid extracts from CSP and CSA showed statistically equal values to those of MgCl_2_ and GDL (*p *<* *0.05); from 100 g of raw soybeans, the tofu samples yielded ranging from 228.9 to 244.8 g (wet basis), and the protein coagulation rates ranged from 88.2% to 90.9%. The tofu yields obtained after pressing have been reported that ranged from ca. 151 to 249 g per 100 g raw soybean (wet basis) (Kim, Choi, Noh, Cho, & Suh, 2007; Lu et al., 1980; Yoon & Kim, 2007).

### 3.5 The textural properties of the tofu samples

The textural properties of the tofu samples were characterized by the mechanical determination of their hardness, springiness, cohesiveness, and gumminess (Table 5). Coagulant types and concentrations are the most crucial factors for the textural property of tofu (Johnson & Wilson, 1984). In some cases, a softer tofu texture was desirable with the use of GDL or acetic acid; the addition of divalent ions increased the hardness of tofu, because it increased ionic binding (Chang et al., 2003; Kim et al., 2007). However, regarding the hardness and springiness, there were no significant differences according to the coagulant types (*p *<* *0.05); only the hardness of the tofu with 0.08% acetic acid was slightly lower than the others. In both the cohesiveness and gumminess, the tofu samples with CaSO_4_, 0.08% acetic acid or CSA‐A1 showed relatively low values. Between MgCl_2_ and all the acetic acid extracts, no significant differences were observed in the two parameters (*p *<* *0.05).

**Table 5:** The textural properties of the tofu samples coagulated with the crab shell extracts or commercial coagulants

Coagulant	Hardness (g)	Springiness	Cohesiveness	Gumminess
MgCl_2_ a	318.1 ± 82.4^ns,^ d	1.27 ± 0.28^ns^	0.75 ± 0.06^a,^ e	228.8 ± 44.7^a^
CaSO_4_ a	269.7 ± 34.7	0.91 ± 0.09	0.51 ± 0.07^bc^	130.2 ± 11.5^bc^
GDL^a^	291.3 ± 42.2	1.06 ± 0.33	0.68 ± 0.05^abc^	181.8 ± 28.8^abc^
Acetic acid	260.7 ± 49.8	0.93 ± 0.12	0.49 ± 0.09^c^	121.8 ± 17.0^c^
CSP‐A1^b^	273.2 ± 43.5	0.95 ± 0.05	0.52 ± 0.12^bc^	136.0 ± 13.4^bc^
CSP‐A3^b^	294.4 ± 33.1	1.12 ± 0.17	0.71 ± 0.07^ab^	199.5 ± 17.1^a^
CSP‐A5^b^	304.5 ± 13.6	0.98 ± 0.07	0.72 ± 0.04^ab^	220.2 ± 10.2^a^
CSA‐A1^c^	311.9 ± 17.3	1.01 ± 0.04	0.69 ± 0.06^abc^	219.2 ± 12.3^a^
CSA‐A3^c^	309.3 ± 42.2	0.99 ± 0.06	0.63 ± 0.07^abc^	190.1 ± 19.4^ab^
CSA‐A5^c^	283.4 ± 48.9	1.16 ± 0.38	0.63 ± 0.08^abc^	198.6 ± 21.7^a^

Data expressed as the mean ± *SD* in quintuplicate determinations.

a Commercial coagulants.

b The extracts from CSP with 1%–5% acetic acid.

c The extracts from CSA with 1%–5% acetic acid.

d No significant difference in the vertical column (*p *<* *0.05).

e The different small letters indicate significantly different values in each the vertical column (*p *<* *0.05).

### 3.6 The sensorial acceptability of the tofu samples

Table 6 represents the sensorial acceptability of the tofu samples with the six acetic acid extracts or commercial coagulants. The acceptability test was conducted using a nine‐point hedonic scale, and all the panelists agreed that less values than 4.5 point on the overall acceptance were considered unacceptable. In appearance, the lowest value was observed in the tofu with CaSO_4_; this might be due to the smaller lump formed and high turbidity of the contained liquid compared to those of the other tofu samples. Although no significant differences were observed in the color and texture among all the tofu samples (*p *<* *0.05), the results in the texture, except for CSP‐A1, were matched well with the mechanical results of the cohesiveness and gumminess. In both the flavor and taste, the tofu with 0.08% acetic acid received significantly lower points than those of all the commercial coagulants due to its sour flavor (*p *<* *0.05); this phenomenon was also found in other reports (Lu et al., 1980; No & Meyers, 2004). When the tofu was prepared with the acetic acid extracts, the scores in flavor decreased with increasing the treated acetic acid concentrations. The results for the overall acceptance suggested that the acetic acid extracts CSP‐A3, CSA‐A1, and CSA‐A3 might be acceptable to the consumer, because their scores were statistically equal to those of the commercial coagulants MgCl_2_ and GDL (*p *<* *0.05).

**Table 6:** Sensorial acceptability of the tofu samples coagulated with the crab shell extracts or commercial coagulants

Coagulant	Appearance	Color	Flavor	Taste	Texture	Overall acceptance
MgCl_2_ a	7.7 ± 0.6^a,^ d	7.3 ± 0.6^ns,^ e	7.2 ± 0.4^a^	7.3 ± 1.0^a^	6.7 ± 1.2^ns^	7.1 ± 0.4^a^
CaSO_4_ a	4.3 ± 0.9^b^	6.3 ± 0.5	6.7 ± 0.5^a^	6.3 ± 0.4^a^	5.8 ± 0.6	5.7 ± 0.6^ab^
Gluconic acid^a^	6.6 ± 0.4^ab^	6.7 ± 1.3	6.3 ± 1.1^a^	6.4 ± 0.6^a^	6.1 ± 1.0	6.4 ± 0.6^ab^
Acetic acid	5.4 ± 1.2^ab^	6.6 ± 0.6	3.2 ± 1.2^b^	3.3 ± 1.1^b^	5.4 ± 1.2	3.9 ± 1.3^b^
CSP‐A1^b^	7.3 ± 0.6^a^	7.5 ± 0.5	6.5 ± 1.0^ab^	5.3 ± 0.7^ab^	6.8 ± 0.8	5.5 ± 1.1^ab^
CSP‐A3^b^	7.0 ± 1.0^ab^	7.0 ± 1.0	5.9 ± 0.6^ab^	6.5 ± 0.5^a^	6.3 ± 1.2	6.4 ± 0.9^ab^
CSP‐A5^b^	7.2 ± 0.8^a^	7.0 ± 0.5	4.7 ± 0.5^ab^	5.2 ± 1.1^ab^	7.0 ± 1.0	5.2 ± 1.3^ab^
CSA‐A1^c^	7.8 ± 0.6^a^	7.1 ± 1.6	6.1 ± 1.3^a^	6.4 ± 1.1^a^	7.0 ± 0.4	6.3 ± 1.0^ab^
CSA‐A3^c^	7.5 ± 1.4^a^	7.0 ± 0.7	5.3 ± 1.4^ab^	6.1 ± 0.5^a^	6.8 ± 1.0	6.2 ± 1.1^ab^
CSA‐A5^c^	7.2 ± 1.6^a^	6.5 ± 1.3	4.5 ± 0.9^ab^	4.9 ± 1.0^ab^	6.6 ± 1.2	4.5 ± 0.7^ab^

*Notes*

Point indicate: 1, extreme dislike; 9, like very much.

Data expressed as the mean ± *SD* of 10 panelists (five men and five women, age 20–40).

a Commercial coagulants.

b The extracts from CSP with 1%–5% acetic acid.

c The extracts from CSA with 1%–5% acetic acid.

d The different small letters indicate significantly different values in each the vertical column (*p *<* *0.05).

e No significant difference in each the vertical column (*p *<* *0.05).

## 4 CONCLUSION

In the present study, the effects of the crab shell extracts as a coagulant on the tofu quality were investigated for the economic use of the crab shell waste. As an extraction solvent, the use of 1%–5% acetic acids led to preparing calcium‐rich extracts from the crab shell. The results for the tofu yields, textural properties, and sensorial acceptability demonstrated that the crab shell extracts CSP‐A3, CSA‐A1, and CSA‐A3 can be used as a natural coagulant for tofu making. For the preparation of the crab shell extract, the ashing process might not be necessary because the yields of the extracts based on its ash contents among the above three extracts were not much different. These findings are expected to provide a good way for an economic use of the crab shell waste.

## CONFLICT OF INTEREST

Authors declare no conflict of interest.
