# Supplementary material for: Effects of crab shell extract as a coagulant on the textural and sensorial properties of tofu (soybean curd)
Source: Food Sci Nutr. 2019 Jan 27;7(2):547–53. doi: 10.1002/fsn3.837 (PMC6392879; doi:10.1002/fsn3.837)
Supplement: Supplementary file 1 [file FSN3-7-547-s001.pdf]

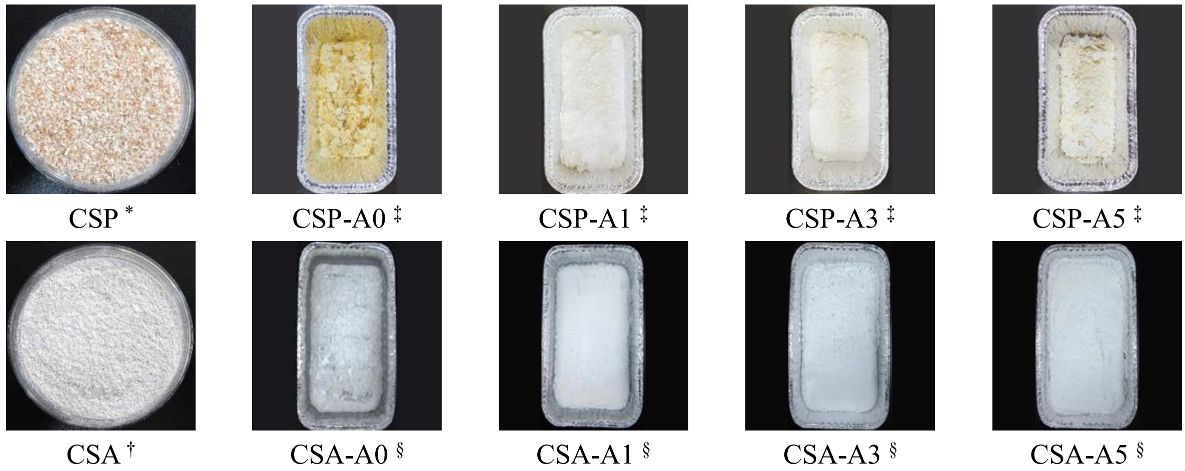

(A) Crab shell powder, ash, and their extracts with 0 to 5 % acetic acid

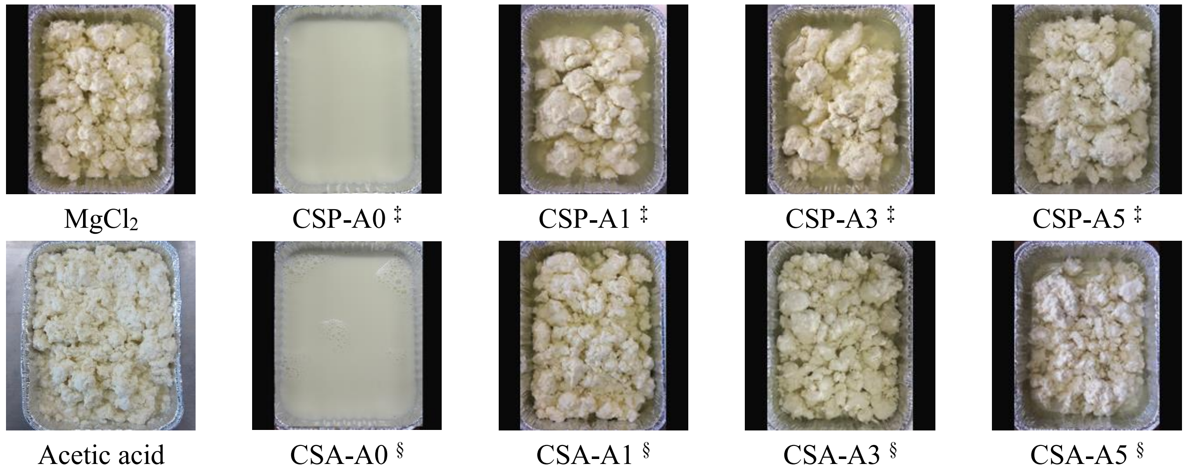

(B) The tofu samples coagulated with the crab shell extracts

Supplementary Fig. 1. Photography of the tofu samples coagulated with the crab shell extracts

[*]Crab shell powder [†]Crab shell ash [‡]The extracts from CSP with 0-5 % acetic acid

[§]The extracts from CSA with 0-5 % acetic acid
